# Effective Identification of Conserved Pathways in Biological Networks Using Hidden Markov Models

**DOI:** 10.1371/journal.pone.0008070

**Published:** 2009-12-07

**Authors:** Xiaoning Qian, Byung-Jun Yoon

**Affiliations:** 1 Department of Computer Science and Engineering, University of South Florida, Tampa, Florida, United States of America; 2 Department of Electrical and Computer Engineering, Texas A&M University, College Station, Texas, United States of America; Fondazione Telethon, Italy

## Abstract

**Background:**

The advent of various high-throughput experimental techniques for measuring molecular interactions has enabled the systematic study of biological interactions on a global scale. Since biological processes are carried out by elaborate collaborations of numerous molecules that give rise to a complex network of molecular interactions, comparative analysis of these biological networks can bring important insights into the functional organization and regulatory mechanisms of biological systems.

**Methodology/Principal Findings:**

In this paper, we present an effective framework for identifying common interaction patterns in the biological networks of different organisms based on hidden Markov models (HMMs). Given two or more networks, our method efficiently finds the top 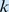 matching paths in the respective networks, where the matching paths may contain a flexible number of consecutive insertions and deletions.

**Conclusions/Significance:**

Based on several protein-protein interaction (PPI) networks obtained from the Database of Interacting Proteins (DIP) and other public databases, we demonstrate that our method is able to detect biologically significant pathways that are conserved across different organisms. Our algorithm has a polynomial complexity that grows linearly with the size of the aligned paths. This enables the search for very long paths with more than 10 nodes within a few minutes on a desktop computer. The software program that implements this algorithm is available upon request from the authors.

## Introduction

Recent advances in high-throughput experimental techniques for measuring molecular interactions [1]–[4] have enabled the systematic study of biological interactions on a global scale for an increasing number of organisms [5]. Genome-scale interaction networks provide invaluable resources for investigating the functional organization of cells and understanding their regulatory mechanisms. Biological networks can be conveniently represented as graphs, in which the nodes represent the basic entities in a given network and the edges indicate the interactions between them. Network alignment provides an effective means for comparing the networks of different organisms by aligning these graphs and finding their common substructures. This can facilitate the discovery of conserved functional modules and ultimately help us study their functions and the detailed molecular mechanisms that contribute to these functions. For this reason, there have been growing efforts to develop efficient network alignment algorithms that can effectively detect conserved interaction patterns in various biological networks, including protein-protein interaction (PPI) networks [6]–[20], metabolic networks [7], [12], [21], gene regulatory networks [22], and signal transduction networks [23]. It has been demonstrated that network alignment algorithms can detect many known biological pathways and also make statistically significant predictions of novel pathways.

Network alignment can be broadly divided into two categories, namely, *global alignment*, which tries to find the best coherent mapping between nodes in different networks that covers all nodes; and *local alignment*, which simply tries to detect significant common substructures in the given networks. Typically, the global network alignment problem is formulated as a graph matching problem whose goal is to find the optimal alignment that maximizes a global objective function that simultaneously measures the similarity between the constituent nodes and also between their interaction patterns. This optimization problem can be solved by a number of techniques, such as integer programming [24], spectral clustering [16], [17], and message passing [20]. To cope with the high complexity of the global alignment problem, many algorithms incorporate heuristic techniques, such as greedy extension of high scoring subnetwork alignments and progressive construction of multiple network alignments [9], [15], [17], [19].

There are also many local network alignment algorithms, where examples include PathBLAST [6], NetworkBLAST [10], QPath [11], PathMatch and GraphMatch [12], just to name a few. These algorithms can effectively find conserved substructures with relatively small sizes, but many of them suffer from high computational complexity that makes it difficult to find larger substructures. Furthermore, many algorithms have limited flexibility of handling node insertions and deletions and/or rely on randomized heuristics that may not necessarily yield optimal results. In [18], we introduced an effective framework for local network alignment based on hidden Markov models (HMMs) that can effectively overcome many of these issues. The HMM framework can naturally integrate both the “node similarity” (typically estimated by sequence similarity) and the “interaction reliability” into the scoring scheme for comparing aligned paths, and it can deal with a large class of path isomorphism. Based on the HMM-based framework, we devised an efficient algorithm that can find the optimal homologous pathway for a given query pathway in a PPI network, whose complexity is linear with respect to the network size and the query length, making it applicable to search for long pathways. It was demonstrated that the algorithm can accurately detect homologous pathways that are biologically significant. However, the algorithm in [18] was mainly developed for *querying* pathways in a target network, hence it cannot be directly used for local alignment of general networks.

In this paper, we extend the HMM-based framework proposed in [18] to make it applicable for local alignment of general biological networks. Especially, we focus on the problem of identifying similar pathways that are conserved across two or more biological networks. Based on HMMs, we propose a general probabilistic framework for scoring pathway alignments and present an efficient search algorithm that can find the top 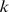 alignments of homologous pathways with the highest scores. The algorithm has polynomial complexity which increases linearly with the length of the aligned pathways as well as the number of interactions in each network. The aligned pathways in a predicted alignment may contain flexible number of consecutive insertions and/or deletions. By combining the high-scoring pathway alignments that overlap with another, we can also detect conserved subnetworks with a general structure. Note that the algorithm can be also used for network querying, by designating one network as the query and another network as the target network.

## Methods

In this section, we present an algorithm for solving the local network alignment problem based on HMMs. For simplicity, we first focus on the problem of aligning two networks, which can be formally defined as follows: Given two biological networks 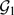 and 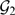 and a specified length 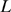, find the most similar pair 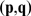 of linear paths, where 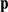 belongs to the network 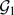 and 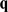 belongs to 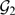, and each of them have 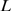 nodes. As we show later, the pairwise network alignment algorithm can be easily extended for aligning multiple networks in a straightforward manner.

### Pairwise Network Alignment

Let 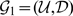 be a graph representing a biological network. We assume that 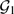 has a set 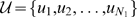 of 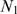 nodes, representing the entities in the network, and a set 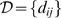 of 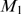 edges, where 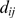 represents the interaction (binding or regulation) between 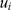 and 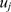. When the network 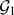 is undirected, we assume that both 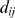 and 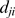 are present in the set 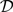 for simplicity. For example, when 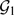 represents a PPI network, 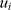 corresponds to a protein, and the edge between 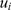 and 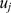 indicates that these proteins can bind to each other. For a pair 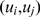 of interacting nodes such that 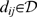, we define their interaction reliability as 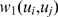. Similarly, let 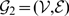 be another graph with 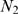 nodes and 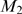 edges, representing a different biological network. We denote the interaction reliability between two nodes 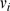 and 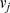 in the graph 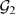 as 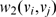. Finally, we denote the similarity between two nodes 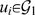 and 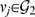 in the respective networks as 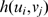, which may be derived using the sequence similarity between two biological entities represented by two nodes as in our experiments.

Our goal is to find the best matching pair of paths 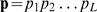 (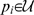) and 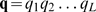 (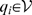) in the respective networks that maximizes a predefined pathway alignment score 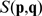. In order to obtain meaningful results, the alignment score 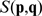 should sensibly integrate the similarity score 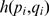 between aligned nodes 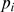 and 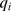 (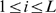), the interaction reliability scores 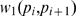 between 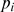 and 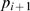 (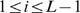) and 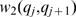 between 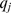 and 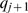 (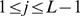), and the penalty for any gaps in the alignment.

**Figure 1:**
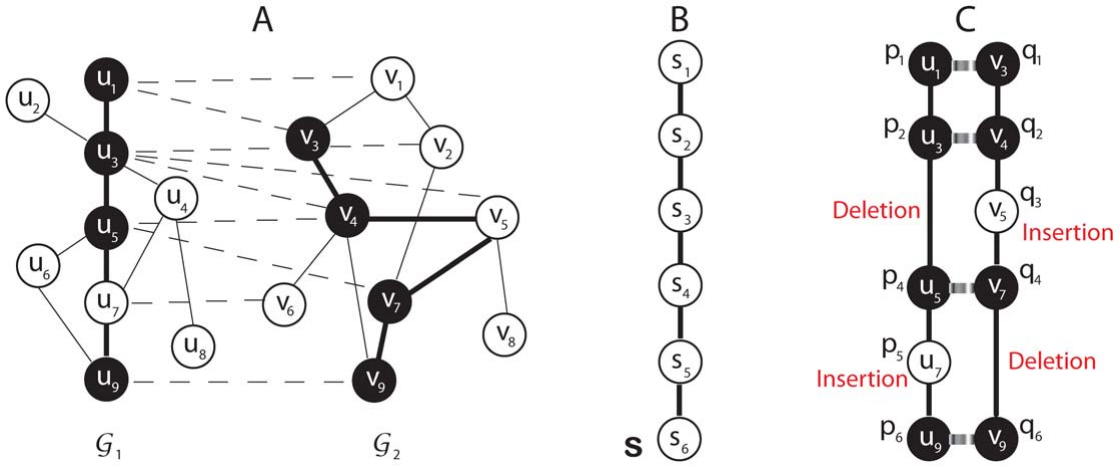
Network representation and alignment. (A) Example of two undirected biological networks 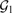 and 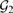. (B) A virtual path 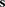 that corresponds to the alignment of best matching paths. (C) The top-scoring alignment between two similar paths 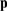 (in 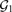) and 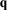 (in 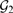).

Figure 1C illustrates an example of an alignment between two similar paths 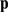 and 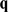, where 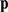 belongs to 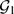 and 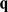 belongs to 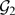 as shown in Fig. 1A. The dashed lines in Fig. 1A that connect two nodes 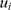 and 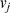 indicate that there exist significant similarities between the connected nodes. In the example shown in Figure 1C, the optimal alignment that maximizes the alignment score 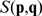 has two gaps at 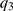 and 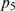. Note that “insertions” and “deletions” are relative terms, and an insertion in 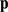 (e.g., 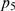) can be viewed as a deletion in the aligned path 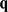, and similarly, an insertion in 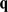 (e.g., 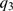) can be viewed as a deletion in 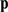.

### Network Representation by HMM

**Figure 2:**
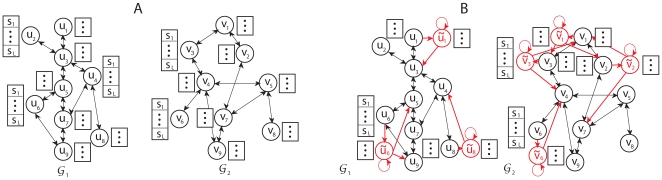
Hidden Markov models for network alignment. (A) Ungapped hidden Markov models (HMMs) for finding the best matching pair of paths. The dots next to the hidden states represent all possible symbols corresponding to virtual nodes in 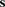 that can be emitted. (B) Modified HMMs that allow insertions and deletions. For simplicity, changes to the HMMs are shown only for the nodes 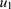, 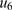, and 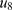 in 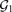; 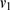, 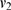, 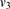, and 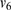 in 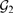.

To define the alignment score 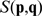, we adopt the hidden Markov model (HMM) formalism. We begin by constructing two HMMs based on the network graphs 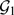 and 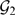. Let us first focus on the construction of HMM for 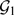. Each node 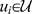 in 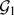 corresponds to a hidden state in the HMM. For convenience, we represent this hidden state using the same notation 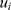. For each edge 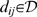 in the graph 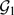, we add an edge from state 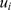 to state 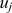 in the HMM. The resulting HMM has an identical structure as the network graph 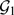. The HMM for 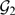 can be constructed in a similar way. Figure 2A illustrates the HMMs that correspond to the network graphs shown in Fig. 1A. In order to find the best matching pairs of paths in the given networks, we define the concept of a “virtual” path 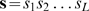 that contains 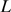 nodes, as shown in Fig. 1B. A node 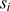 in the virtual path can be viewed as a symbol that is emitted by a pair of hidden states 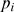 and 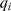 in the respective HMMs. From this point of view, the two HMMs can be regarded as generative models that *jointly* produce (or “emit”) the virtual path 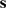, and the underlying state sequence for 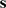 will be a pair of state sequences 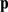 and 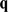 in the respective HMMs. Therefore, the concept of a virtual path can naturally couple a path in 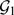 with another in 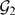, providing a convenient framework for identifying conserved pathways in the original biological networks.

The described HMM-based network representation allows us to naturally integrate the interaction reliability scores and the node similarity scores into an effective probabilistic framework. We first define two mappings 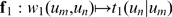 and 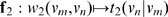, which convert the interaction reliability scores 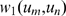 and 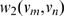 between two nodes in 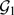 and 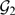 to the following transition probabilities
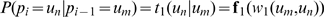
(1)

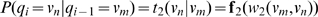
(2)between the corresponding hidden states in the constructed HMMs. The mapping 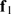 is defined so that (i) 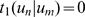 for 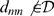, (ii) 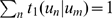 for all 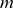, and (iii) 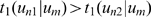 for 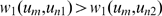. Similarly, the mapping 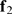 follows the same constraints: (i) 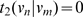 for 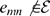, (ii) 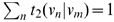 for all 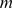, and (iii) 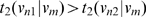 for 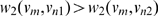. To specify the emission probability of a virtual symbol 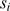 at a pair of hidden states in the two HMMs, we define another mapping 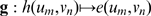 that converts the node similarity score 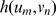 to the following “pairing” probability
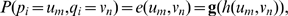
(3)where 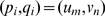 is the pair of underlying hidden states for 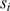. The mapping 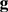 is defined so that (i) 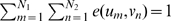 for all possible pairs of 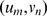, and (ii) 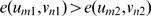 for 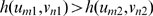.

### Ungapped Alignment

Based on the HMM framework, the problem of finding the best matching pair of paths is transformed into the problem of finding the optimal pair of state sequences in the two HMMs that jointly maximize the observation probability of the virtual path 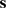. In an ungapped pathway alignment, the underlying state pair 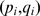 of a virtual symbol 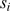 directly corresponds to a pair of aligned nodes in the original networks. We can find the optimal pair of paths in polynomial time by using a dynamic programming algorithm defined in the following, which is conceptually similar to the Viterbi algorithm. We first define 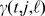 as the log-probability of the most probable pair of paths for a subsequence 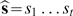 of length 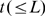, where the underlying states for the virtual symbol 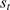 are 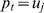 and 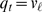. The log-probability 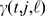 can be recursively computed as follows:
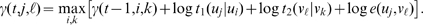
(4)


We repeat the above iterations until 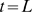. At the end of the iterations, the maximum log-probability of the virtual path 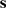 is given by:
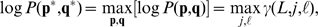
(5)where 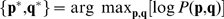 is the optimal pair of state sequences that correspond to the best matching paths in the original biological networks. Once we have computed 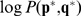, it is straightforward to find 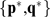 by tracing the recursive equations that led to the maximum log-probability 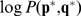. Although the above algorithm only finds the top-scoring pair of paths, we can easily extend it to find the top 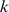 pairs simply by replacing the 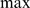 operator by an operator that finds the 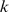 largest scores.

The computational complexity of the above algorithm is 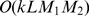 for finding the top 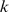 pairs of matching paths, where 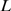 is the length of the aligned paths that we want to find, 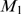 is the number of edges in 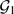, and 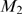 is the number of edges in 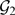. Note that the complexity is linear with respect to all the parameters 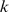, 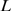, 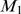, and 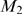.

The log-probability 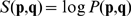 can serve as a good alignment score for the paths 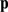 and 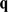 that effectively combines node similarity and interaction reliability. In principle, we can also use non-stochastic emission (pairing) scores 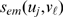 and transition scores 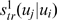 and 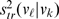 in the recursive equation (4), in place of the log-probabilities 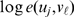, 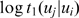, and 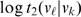, respectively. This will yield a non-stochastic pathway alignment score instead of an observation probability.

As we can see, the concept of the “virtual” path provides an intuitive way of coupling states in two different HMMs. In fact, by taking a closer look at the recursive equation (4), the proposed alignment algorithm can also be viewed as a Markovian walk on a product graph, whose nodes consist of all possible pairs of hidden states in the respective HMMs and the edges between these nodes are determined by the connectivity (or transition probability) between the corresponding states in the HMMs. The algorithm searches for the optimal path (or the top-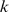 paths) in the product graph that yields the highest score based on the parameters of the given HMMs.

### Alignment with Gaps

To accommodate gaps in the aligned paths 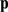 and 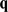, we modify the previous HMMs as follows. First, we add an accompanying state 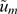 for every state 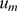 in 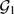, and similarly, we add an accompanying state 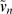 for every state 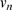 in 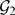. Next, we add an outgoing edge from each state to the corresponding accompanying state. In addition to this, we also add outgoing edges from the accompanying state to all the neighboring states of the original state. To be more precise, 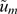 will have an outgoing edge to every 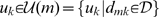, and 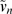 will have an outgoing edge to every 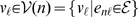. By varying the transition probabilities 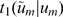 and 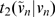, we can control the probabilities of having insertions and/or deletions, and thereby control the “gap penalties” in a pathway alignment. We adjust the outgoing transition probability from 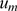 so that 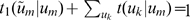; and for the outgoing transition probability from 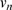 so that 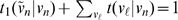. We can also control the probabilities of having *consecutive* insertions or deletions by adjusting the probabilities 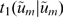 and 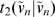 for making self-transitions at either 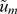 or 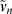. The outgoing transition probabilities 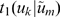 from an accompanying state 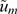 are chosen so that they are proportional to 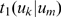 and satisfy 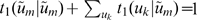. The transition probabilities in 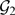 can be chosen in a similar manner. The structures of the modified HMMs are depicted in Fig. 2B. Note that, in a gapped alignment, the matching paths (or state sequences) 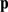 and 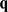 will still contain 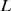 nodes each, and the only difference from an ungapped alignment is that the paths may now contain one or more accompanying nodes which represent gaps. The proposed framework does not impose any restriction on the number of gaps and their locations in the pathway alignment.

In order to find the optimal pair of paths (and their alignment) that maximize the pathway alignment score, we can apply the same dynamic programming algorithm described in the previous section. The retrieved paths can contain any of the hidden states 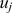 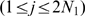 and 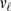 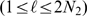 in the modified HMMs, where we define 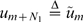 and 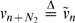 for notational convenience. The optimal paths 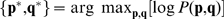 is the best matching pair of paths from two networks, and they may now contain insertions and/or deletions. As before, if we want to find the top 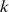 pairs instead of a single top-scoring pair, we can simply replace the 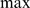 operator by an operator that finds the 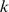 largest scores. Note that the computational complexity of the algorithm is 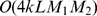, which is still linear with respect to all the parameters.

### Extension to Multiple Networks

It is straightforward to extend the described pairwise network alignment algorithm for aligning multiple networks. Without loss of generality, we only consider the extension to the alignment of three networks. Given three network graphs 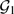, 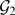, and 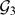, we construct the corresponding HMMs based on their structures. We again use the concept of virtual paths, and now we assume that a virtual path 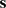 is jointly emitted by these three HMMs. The emission of a virtual symbol 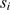 is now governed by a pairing probability 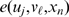 of three hidden states 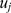, 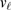, and 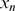 that belong to the HMMs that correspond to 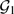, 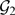, and 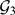, respectively. We can find the best matching paths based on the following recursive equation:
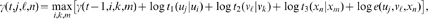
(6)where 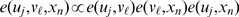 is assumed for simplicity. We repeat the above iterations until we reach 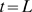 and compute the maximum log-probability as follows:
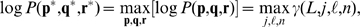
(7)where 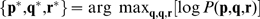 corresponds to the set of best matching paths in the three networks.

### Implementation of the Alignment Algorithm

It should be noted that although we fix the length of the virtual path to 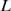, we can in fact find any top-scoring alignment with a shorter length 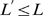, since we store all the alignment scores for shorter alignments while running the dynamic programming algorithm. The recursive equations in (4) and (6) do not restrict multiple occurrence of the same node in the final pathway alignment. However, when it is desirable to avoid such multiple occurrence, we can easily incorporate a “look-back” step into each iteration in order to prevent adding a node that is already included in the (intermediate) alignment. As this requires tracing the intermediate optimal (or top 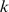) alignment, the computational complexity of the recursive equations (4) and (6) with a “look-back” step will be increased in proportion to the length of the intermediate alignment.

In order to obtain more general subnetwork alignments, not just alignments of linear paths, we can combine the overlapping paths among the top 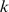 retrieved pairs of paths. The edges that are already contained in the constructed subnetwork alignment (which correspond to the conserved molecular interactions in the biological networks) are then removed from the HMMs, and we run the dynamic programming algorithm again to find another subnetwork alignment that does not overlap with the retrieved subnetworks. By repeating this “search and peel-off” process, we can effectively find diverse subnetwork regions that are conserved in the given networks.

The memory complexity of the proposed algorithm is 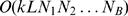 for finding the top 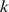 pathway alignments for 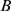 networks. Although the required amount of memory increases only linearly with respect to each parameter, it can still make the algorithm infeasible when we want to align multiple number of large networks. To overcome this problem, we may assign non-zero pairing probabilities 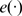 to a set of nodes (in the respective networks) only if every pair in this set has considerable node similarity that exceeds a certain threshold. Assuming that there are 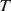 sets of nodes that satisfy this condition, we only need to consider these 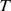 possible node alignments, in which case the overall memory complexity reduces to 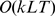. Since 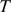 is often much smaller than 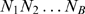, this scheme can save significant amount of memory, thereby making the algorithm feasible.

## Results

To demonstrate the effectiveness of the HMM-based network alignment algorithm, we carried out the following experiments. First, we used our algorithm to align two pairs of small synthetic networks that were used to validate the network alignment algorithm proposed in [24]. Second, we used the proposed algorithm for finding putative pathways in the fruit fly PPI network that look similar to known human pathways. Finally, we applied the algorithm for aligning microbial PPI networks to assess its ability to find conserved functional modules.

### Aligning Synthetic Networks

To illustrate the potential capability of aligning different types of molecular networks, we first tested our algorithm using two small synthetic examples, which include a pair of undirected networks and another pair of directed networks. These examples were obtained from the tutorial files in the PathBLAST plugin of software Cytopscape (version 1.1, http://www.cytoscape.org/plugins1.php) and they were used for the validation of a network alignment algorithm called MNAligner [24].

#### HMM parameterization

For aligning the synthetic networks, we parameterized the HMMs as follows. We set the transition scores 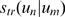 directly based on the “adjacent matrices” given in [24], which contain the interaction scores between two nodes in the respective networks. Every interaction score takes a value between 0 and 1, hence we can view it as the “interaction probability”. We took the logarithm of this interaction probability as the transition score 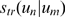. When there is no interaction between two nodes, we have 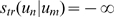. This keeps the HMM from making a direct transition from a state 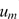 to a non-relevant state 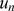, thereby preventing the inclusion of irrelevant protein interactions that do not have any biological support in the network. Similarly, we obtained the emission scores 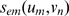 by taking the logarithm of the similarity scores between nodes given by the “similarity matrices” in [24]. The adjacent matrices and the similarity scores for the two examples can be found in the Supporting Information S1.

#### Example 1: Aligning undirected networks

**Figure 3:**
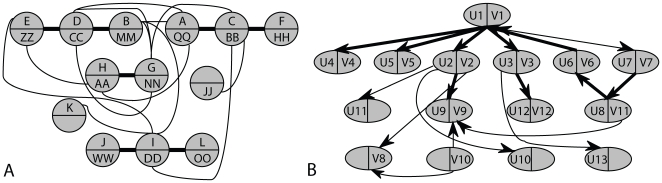
The alignment results for synthetic networks. (A) Undirected networks; (B) Directed networks.

We first used our algorithm for aligning a pair of undirected networks. To compare the alignment results with the results obtained by MNAligner [24], we looked for the top 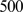 alignments without gaps, where the length of the virtual path was set to 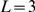. By incorporating “look-back” steps into our dynamic programming algorithm, we restricted the multiple occurrence of the same node pair in the obtained pathway alignment. The top-scoring pathway alignment obtained from our algorithm was 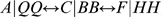, which is identical to the optimal alignment identified by both PathBLAST [6] and MNAligner [24]. Unlike PathBLAST, the proposed HMM-based algorithm and the MNAligner both keep the natural order of the nodes in the original networks. We also noticed that the paths 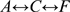 and 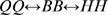 can be aligned with several other potential similar paths in the corresponding networks from the top 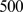 aligned results. After removing the interactions included in the top-scoring alignment, we searched for the next top-scoring alignment. This returned the alignment 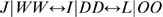, which was also ranked as the second best alignment by MNAligner [24]. Repeating the experiment after removing this alignment returned 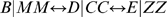 as the third best alignment. This is different from the alignment 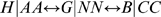 that was found by MNAligner, which got a lower score in our experiment. We noted that the alignment 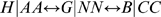 is not as significant as the three alignments that we found, as 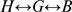 can be aligned with many other paths with the same alignment score. By repeating the above experiments and combining the pathway alignment results, we obtained the global network alignment illustrated in Fig. 3A, where a bold line represents that the corresponding edges in the respective networks are matched, whereas a thin line indicates a mismatch. These results show that the HMM-based method can effectively identify the top matching paths in different undirected networks, and it yields better results with higher alignment scores integrating both node similarity and interaction probability compared to PathBLAST and MNAligner for this purpose.

#### Example 2: Aligning directed networks

Without any modification, our algorithm can also be used for aligning directed networks. We demonstrate this by using the second example that contains a pair of small directed networks. In this experiment, we set the length of the virtual path to 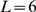, which is the length of the longest path in these two networks. As there are fewer legitimate paths in these networks, we only looked for the top 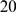 aligned pairs of paths. The obtained pathway alignments were combined to get the global network alignment shown in Fig. 3B. The alignment results were similar to those obtained by MNAligner [24], except that we found fewer aligned nodes and edges. This is natural since there exist only a few similar pairs of nodes in the given networks (see Supporting Information S1) and as our algorithm focuses on finding the best local alignments instead of a global alignment. Note that, unlike PathBLAST, which finds path alignments based on several heuristics, the proposed algorithm can find the mathematically optimal path alignment for the given networks.

### Aligning Annotated Pathways with PPI Networks

#### HMM parameterization

The proposed algorithm can also be used for identifying putative pathways in a new biological network, which look similar to known pathways. To demonstrate this, we used our algorithm to search for human signaling pathways in the fruit fly PPI network. In order to compare the search results with those of the network querying algorithm in [18], the HMMs were parameterized according to the non-stochastic scoring scheme in [18] as we describe in the following. The transition score 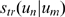 was set to 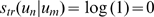 in the presence of interaction between the proteins that correspond to 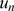 and 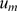, and it was set to 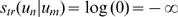 in the absence of any interaction. To allow gaps in alignments, the transition score from a state 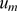 to its accompanying state 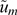 was set to 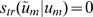, and we set the self-transition score at 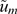 to 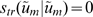 to allow consecutive gaps. Furthermore, the score for making a transition from 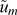 to a regular state 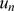 was set to 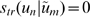 for 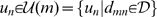 and 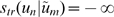 for 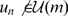. The emission score 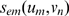 for two proteins 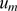 and 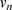 in different networks (where the query network is simply a linear path in this case) was computed based on their sequence similarity. For each protein pair 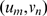, we computed its E-value using the PRSS routine in the FASTA package [25], [26], which is known to yield more accurate E-values compared to BLASTP [27]. We regarded a protein pair 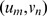 as a “match” if its E-value 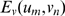 was below a threshold 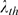. Otherwise, we regarded the pair as a “mismatch”, which implies that the proteins do not bear significant similarity. Based on this criterion, we set the emission score 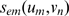 as follows:
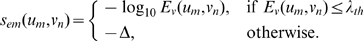
(8)The value 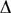 can be viewed as the mismatch penalty, and is selected so that 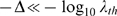. We set the insertion and deletion penalty also to 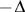. Finally, since two accompanying states cannot be paired with each other, we set 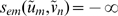.

#### Querying human pathways in the fruit fly PPI network

We first obtained the PPI network of *Drosophila melanogaster* from the Database of Interacting Proteins (DIP) [28] and constructed the “target HMM”. Then we constructed a “query HMM” for the human hedgehog signaling pathway and another query HMM based on the human MAP kinase pathway. When constructing the query HMMs, we regarded each signaling pathway as a “directed network” with a linear structure, instead of a “sequence of proteins” as in [18]. The similarity threshold was set to 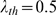 and the gap penalty was set to 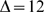, as in [18]. The constructed query HMMs were then used to search for matching paths in the target HMM. Despite the generality and the different implementation of the proposed algorithm, the top pathways retrieved by the proposed algorithm agree with the predictions in [18], which is the direct consequence of the mathematical optimality of both methods. For the human hedgehog signaling pathway lhh–Ptch–Smo–Stk36–Gli, the top-scoring pathway in the *D. melanogaster* network agreed well with the putative *D. melanogaster* hedgehog signaling pathway reported in the KEGG database [29]. In fact, the best aligned path in the fruit fly network contained shh–ptc–Smo–fu–ci, which is identical to the core portion of the putative fly hedgehog signaling pathway (http://www.genome.jp/dbget-bin/get_pathway?org_name=dme&mapno=04340) in the KEGG database [29]. The query result of the human MAP kinase pathway Egfr–drk–Sos–Ras85D–ph1–Mekk1–ERKA was also biologically significant, and the seven proteins in the retrieved pathway matched exactly with the proteins in the putative fruit fly MAP kinase pathway (http://www.genome.jp/dbget-bin/get_pathway?org_name=map&mapno=04010) reported in KEGG. These results compare favorably to the results obtained by one of the state-of-the-art algorithms [11], where they found two identical proteins in the putative fly hedgehog signaling pathway and five proteins in the putative fly MAPK pathway.

### Aligning Microbial PPI Networks

In order to validate the accuracy of our algorithm for predicting functional modules that are conserved in different organisms, we performed additional experiments using three microbial PPI networks obtained from [9]. In our experiments, we performed a pairwise alignment between the *E. coli* and the *C. crescentus* networks as well as a pairwise alignment between the *E. coli* and the *S. typhimurium* networks. We assessed the accuracy of our algorithm based on the consistency of the *KEGG ortholog (KO) group* annotations [29] of the aligned proteins. In order to measure the consistency of KO group annotations, we computed the specificity of the predictions based on a similar methodology that was used in [14]. To compute this measure, we first remove all the aligned protein pairs that do not have complete KO annotations, and then compute the total number of annotated protein pairs. An annotated protein pair is regarded as being *correct* if both proteins have the same KO group annotations, and *incorrect* if the annotations do not agree. The specificity is defined as the ratio of the number of “correct” protein pairs among all annotated protein pairs.

For this experiment, the parameters of the HMMs have been chosen as follows. First, the transition scores 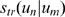 have been obtained by taking the logarithm of the protein interaction probabilities in the microbial networks, which had been assigned by the SRINI algorithm [30]. The emission scores 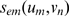 have been computed based on the sequence similarity between the proteins 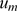 and 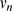, as in the previous section, where the protein similarities have been estimated based on the BLASTP hit scores between protein pairs provided in [9].

**Figure 4:**
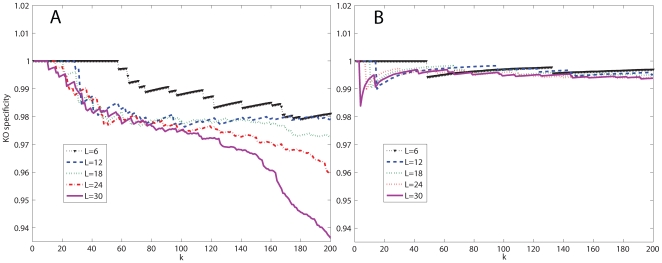
Functional specificity for microbial network alignment. The cumulative specificity of the top 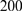 aligned pathways obtained from (A) the pairwise alignment between *E. coli* and *C. crescentus* networks; and (B) the pairwise alignment between *E. coli* and *S. typhimurium* networks.

Based on the constructed HMMs, we used our algorithm to find the top-scoring pathway alignment with gaps. At each iteration, we looked for the top aligned pair of paths, stored the alignment, and removed the interactions included in the alignment from the respective networks for the next iteration. By repeating this iteration, we found 200 high-scoring path alignments. This experiment has been repeated with varying virtual path length: 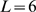, 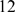, 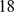, 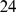, and 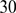. In all our experiments, we disallowed multiple occurrence of identical protein pairs and set the gap/mismatch penalty to 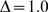. For each experiment, we computed the cumulative specificity for the top 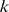 alignments, which is given by
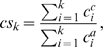
(9)where 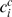 is the total number of correctly aligned protein pairs in the top 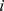 alignments, and 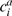 is the total number of annotated protein pairs also in the top 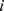 alignments. The result from the pairwise alignment of the *E. coli* and the *C. crescentus* networks is shown in Fig. 4A, and the result from the alignment of the *E. coli* and the *S. typhimurium* networks is shown in Fig. 4B. As we can see in both Fig. 4A and Fig. 4B, the cumulative specificity 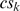 generally decreases when we increase the alignment length 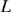. This is expected since the algorithm tends to recruit more protein pairs in the alignment if we increase 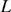. Furthermore, 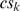 generally decreases if we increase 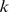. This is natural, since alignments with lower scores correspond to less conserved pathways with larger variations. Although it is difficult to directly compare our results with those reported in [14], it is still worth to note that the cumulative specificity (for the top 200 alignments) of the proposed HMM-based algorithm is higher than the specificity of the alignment algorithm Græmlin 2.0 [14], for both pairwise network alignments. These results clearly indicate that our HMM-based algorithm can produce accurate network alignments that are biologically meaningful.

Further analysis of the predicted alignments led to a number of interesting observations. For example, the alignment of *E. coli* and *C. crescentus* networks and the alignment of *E. coli* and *S. typhimurium* networks both detected conserved DNA replication modules. The module contained components of the primosome (dnaA, gyrA, gyrB), subunits of topoisomerase IV (parC, parE), and a subunit of DNA polymerase III (dnaN). These protein families are all known to be involved in DNA replication. We also found other interesting conserved modules, which include both large and small subunits of ribosomal protein complexes (rplA, rplB, rplC, rplE, rplK, rplP; and rpsA, rpsB, rpsC, rpsE, rpsG, rpsK); DNA-directed RNA polymerase complex containing rpoA, rpoB, rpoC, and other subunits; the citrate cycle (TCA cycle) containing 2-oxoglutarate dehydrogenase E1 component (sucA, sucB) and succinyl-CoA synthetase (sucC, sucD); NADH dehydrogenase I (nuoA, nuoB, nuoC, nuoF, nuoH, nuoI, nuoL, nuoM), which is a part of the oxidative phosphorylation pathway; nitrate reductase 1 (with narG, narH, narI, and narJ); and a portion of the bacterial secretion system (with secA, secD, secY).

## Discussion

In this paper, we proposed an HMM-based network alignment algorithm that can be used for finding conserved pathways in two or more biological networks. The HMM framework and the proposed alignment algorithm has a number of important advantages compared to other existing local network alignment algorithms. First of all, despite its generality, the proposed algorithm is very simple and efficient. In fact, the alignment algorithm based on the proposed HMM framework is a variant of the Viterbi algorithm. As a result, it has a very low polynomial computational complexity, which grows only linearly with respect to the length of the identified pathways and the number of edges in each network. This makes it possible to find conserved pathways with more than 10 nodes in networks with thousands of nodes and tens of thousands of interactions within a few minutes on a personal computer. Furthermore, the HMM-based framework can handle a large class of path isomorphism, which allows us to find pathway alignments with any number of gaps (node insertions and deletions) at arbitrary locations. In addition to this, the proposed framework is very flexible in choosing the scoring scheme for pathway alignments, where different penalties can be used for mismatches, insertions and deletions. We can also assign different penalties for gap opening and gap extension, which can be convenient when comparing networks that are remotely related to each other. Another important advantage of the proposed framework is that it allows us to use an efficient dynamic programming algorithm for finding the mathematically optimal alignment. Considering that many available algorithms rely on heuristics that cannot guarantee the optimality of the obtained solutions, this is certainly a significant merit of the HMM-based approach. Although the mathematical optimality does not guarantee the biological significance of the obtained solution, it can certainly lead to more accurate predictions if combined with a realistic scoring scheme for assessing pathway homology. As demonstrated in our experiments, the proposed algorithm yields accurate and biologically meaningful results both for querying known pathways in the network of another organism and also for finding conserved functional modules in the networks of different organisms. Finally, the HMM-based framework presented in this paper can be extended for aligning multiple networks. While many current multiple network alignment algorithms adopt a progressive approach for comparing multiple networks [9], [14]–[17], our HMM-based framework provides a potential way to simultaneously align multiple networks to find the optimal set of conserved pathways with maximum alignment score.

For future research, we plan to evaluate the performance of our HMM-based algorithm more extensively by investigating the consistency of the predicted alignments based on other available functional annotations, including the gene ontology (GO) annotations [31]. It would be also beneficial to develop a more elaborate scoring scheme that integrates additional information, such as the GO annotations and the KO group annotations, to obtain more reliable alignment results. Finally, we are currently working on simultaneous multiple network alignment based on the HMM framework, where the goal is to construct a scalable multiple alignment algorithm that yields network alignments with higher fidelity.

## Supporting Information

Supporting Information S1 (0.06 MB PDF)
