# Supplementary material for: Effective Identification of Conserved Pathways in Biological Networks Using Hidden Markov Models
Source: PLoS One. 2009 Dec 7;4(12):e8070. doi: 10.1371/journal.pone.0008070 (PMC2782142; doi:10.1371/journal.pone.0008070)
Supplement: Supporting Information S1 — (0.06 MB PDF) [file pone.0008070.s001.pdf]

This supplementary file provides the supplementary materials for the manuscript **“Effective Identification of Conserved Pathways in Biological Networks Using Hidden Markov Models”**, including the relevant information about the synthetic examples in our experiments, which we have obtained from the tutorial files in the PathBLAST [1] plugin of software Cytopscape [2] (version 1.1) and they were used for the validation of a network alignment algorithm called MNAligner [3].

*** Note:** The order of the references in this file is *not* identical to the order in the manuscript. The list of references cited in this file can be found on the last page.

Adjacent matrices and similarity matrices for two synthetic examples

Example 1

1. Adjacent matrix for the first undirected network to align (The ordered labels for the nodes in this network is – ‘A’, ‘B’, ‘C’, ‘D’, ‘E’, ‘F’, ‘G’, ‘H’, ‘I’, ‘J’, ‘K’, ‘L’):

$$
\begin{pmatrix}
0 & 0.10 & 0.70 & 0.01 & 0 & 0 & 0 & 0 & 0 & 0 & 0 & 0 \\
0.10 & 0 & 0 & 0.30 & 0 & 0 & 0.01 & 0 & 0.02 & 0 & 0 & 0 \\
0.70 & 0 & 0 & 0 & 0 & 0.20 & 0.01 & 0 & 0 & 0 & 0 & 0 \\
0.01 & 0.30 & 0 & 0 & 0.20 & 0 & 0.01 & 0 & 0 & 0 & 0 & 0 \\
0 & 0 & 0 & 0.20 & 0 & 0 & 0 & 0 & 0.01 & 0 & 0 & 0 \\
0 & 0 & 0.20 & 0 & 0 & 0 & 0 & 0 & 0 & 0 & 0 & 0 \\
0 & 0.01 & 0.01 & 0.01 & 0 & 0 & 0 & 0.70 & 0 & 0 & 0 & 0 \\
0 & 0 & 0 & 0 & 0 & 0 & 0.70 & 0 & 0 & 0 & 0 & 0 \\
0 & 0.02 & 0 & 0 & 0.01 & 0 & 0 & 0 & 0 & 0.30 & 0.01 & 0.60 \\
0 & 0 & 0 & 0 & 0 & 0 & 0 & 0 & 0.30 & 0 & 0 & 0 \\
0 & 0 & 0 & 0 & 0 & 0 & 0 & 0 & 0.01 & 0 & 0 & 0 \\
0 & 0 & 0 & 0 & 0 & 0 & 0 & 0 & 0.60 & 0 & 0 & 0
\end{pmatrix}.
$$

2. Adjacent matrix for the second undirected network to align (The ordered labels for the nodes in this network is – ‘AA’, ‘BB’, ‘CC’, ‘DD’, ‘HH’, ‘MM’, ‘ZZ’, ‘NN’, ‘QQ’, ‘JJ’, ‘OO’, ‘WW’):

$$
\begin{pmatrix}
0 & 0 & 0 & 0 & 0 & 0 & 0.01 & 0.20 & 0.10 & 0 & 0 & 0 \\
0 & 0 & 0 & 0.01 & 0.70 & 0 & 0 & 0 & 0.70 & 0.01 & 0 & 0 \\
0 & 0 & 0 & 0 & 0 & 0.02 & 0.20 & 0.10 & 0 & 0 & 0 & 0 \\
0 & 0.01 & 0 & 0 & 0 & 0 & 0 & 0 & 0 & 0 & 0.10 & 0.01 \\
0 & 0.70 & 0 & 0 & 0 & 0 & 0 & 0 & 0 & 0 & 0 & 0 \\
0 & 0 & 0.02 & 0 & 0 & 0 & 0 & 0 & 0 & 0 & 0 & 0 \\
0.01 & 0 & 0.20 & 0 & 0 & 0 & 0 & 0 & 0 & 0 & 0 & 0 \\
0.20 & 0 & 0.10 & 0 & 0 & 0 & 0 & 0 & 0 & 0 & 0 & 0 \\
0.10 & 0.70 & 0 & 0 & 0 & 0 & 0 & 0 & 0 & 0 & 0 & 0 \\
0 & 0.01 & 0 & 0 & 0 & 0 & 0 & 0 & 0 & 0 & 0 & 0 \\
0 & 0 & 0 & 0.10 & 0 & 0 & 0 & 0 & 0 & 0 & 0 & 0 \\
0 & 0 & 0 & 0.01 & 0 & 0 & 0 & 0 & 0 & 0 & 0 & 0
\end{pmatrix}.
$$

3. Similarity matrix between the nodes from two undirected networks to align:

$$\begin{pmatrix}
0.1 & 0.1 & 0.1 & 0.8 & 0.5 & 0.1 & 0.1 & 0.8 & 0.8 & 0.1 & 0.1 & 0.1 \\
0.1 & 0.1 & 0.8 & 0.1 & 0.1 & 0.1 & 0.8 & 0.1 & 0.1 & 0.1 & 0.1 & 0.1 \\
0.1 & 0.8 & 0.1 & 0.1 & 0.1 & 0.1 & 0.1 & 0.1 & 0.1 & 0.1 & 0.1 & 0.1 \\
0.1 & 0.8 & 0.1 & 0.1 & 0.1 & 0.1 & 0.1 & 0.1 & 0.1 & 0.1 & 0.1 & 0.1 \\
0.1 & 0.8 & 0.1 & 0.1 & 0.1 & 0.1 & 0.8 & 0.1 & 0.1 & 0.1 & 0.8 & 0.1 \\
0.1 & 0.1 & 0.1 & 0.1 & 0.8 & 0.1 & 0.1 & 0.8 & 0.1 & 0.1 & 0.1 & 0.1 \\
0.8 & 0.1 & 0.1 & 0.1 & 0.1 & 0.1 & 0.1 & 0.1 & 0.1 & 0.1 & 0.8 & 0.1 \\
0.8 & 0.1 & 0.1 & 0.1 & 0.1 & 0.1 & 0.1 & 0.1 & 0.1 & 0.1 & 0.1 & 0.1 \\
0.1 & 0.1 & 0.1 & 0.8 & 0.1 & 0.8 & 0.8 & 0.1 & 0.1 & 0.1 & 0.1 & 0.8 \\
0.1 & 0.1 & 0.1 & 0.8 & 0.1 & 0.1 & 0.1 & 0.1 & 0.1 & 0.1 & 0.1 & 0.8 \\
0.8 & 0.1 & 0.1 & 0.1 & 0.1 & 0.8 & 0.1 & 0.1 & 0.8 & 0.1 & 0.1 & 0.8 \\
0.1 & 0.1 & 0.8 & 0.8 & 0.1 & 0.1 & 0.1 & 0.1 & 0.1 & 0.1 & 0.8 & 0.8
\end{pmatrix}.$$

Example 2

1. Adjacent matrix for the first directed network to align (The ordered labels for the nodes in this network is – ‘U1’, ‘U2’, ‘U3’, ‘U4’, ‘U5’, ‘U6’, ‘U7’, ‘U8’, ‘U9’, ‘U10’, ‘U11’, ‘U12’, ‘U13’):

$$\begin{pmatrix}
0 & 1 & 1 & 1 & 1 & 0 & 1 & 0 & 0 & 0 & 0 & 0 & 0 \\
0 & 0 & 0 & 0 & 0 & 0 & 0 & 0 & 1 & 1 & 1 & 0 & 0 \\
0 & 0 & 0 & 0 & 0 & 0 & 0 & 0 & 0 & 0 & 0 & 1 & 1 \\
0 & 0 & 0 & 0 & 0 & 0 & 0 & 0 & 0 & 0 & 0 & 0 & 0 \\
0 & 0 & 0 & 0 & 0 & 0 & 0 & 0 & 0 & 0 & 0 & 0 & 0 \\
1 & 0 & 0 & 0 & 0 & 0 & 0 & 0 & 0 & 0 & 0 & 0 & 0 \\
0 & 0 & 0 & 0 & 0 & 0 & 0 & 1 & 0 & 0 & 0 & 0 & 0 \\
0 & 0 & 0 & 0 & 0 & 1 & 0 & 0 & 0 & 0 & 0 & 0 & 0 \\
0 & 0 & 0 & 0 & 0 & 0 & 0 & 0 & 0 & 0 & 0 & 0 & 0 \\
0 & 0 & 0 & 0 & 0 & 0 & 0 & 0 & 0 & 0 & 0 & 0 & 0 \\
0 & 0 & 0 & 0 & 0 & 0 & 0 & 0 & 0 & 0 & 0 & 0 & 0 \\
0 & 0 & 0 & 0 & 0 & 0 & 0 & 0 & 0 & 0 & 0 & 0 & 0 \\
0 & 0 & 0 & 0 & 0 & 0 & 0 & 0 & 0 & 0 & 0 & 0 & 0
\end{pmatrix}.$$

2. Adjacent matrix for the second directed network to align (The ordered labels for the nodes in this

network is – ‘V1’, ‘V2’, ‘V3’, ‘V4’, ‘V5’, ‘V6’, ‘V7’, ‘V8’, ‘V9’, ‘V10’, ‘V11’, ‘V12’):

$$\begin{pmatrix} 0&1&1&1&1&0&1&0&0&0&0&0\\ 0&0&0&0&0&0&0&1&1&0&0&0\\ 0&0&0&0&0&0&0&0&0&0&0&1\\ 0&0&0&0&0&0&0&0&0&0&0&0\\ 0&0&0&0&0&0&0&0&0&0&0&0\\ 1&0&0&0&0&0&0&0&0&0&0&0\\ 0&0&0&0&0&0&0&0&0&0&1&0\\ 0&0&0&0&0&0&0&0&0&0&0&0\\ 0&0&0&0&0&0&0&0&0&0&0&0\\ 0&0&0&0&0&0&0&1&1&0&0&0\\ 0&0&0&0&0&1&0&0&1&0&0&0\\ 0&0&0&0&0&0&0&0&0&0&0&0 \end{pmatrix}.$$

3. Similarity matrix between the nodes from two directed networks to align:

$$\begin{pmatrix} 1&0&0&0&0&0&0&0&0&0&0&0\\ 0&1&0&0&0&0&0&0&0&0&0&0\\ 0&0&1&0&0&0&0&0&0&0&0&0\\ 0&0&0&1&0&0&0&0&0&0&0&0\\ 0&0&0&0&1&0&0&0&0&0&0&0\\ 0&0&0&0&0&1&0&0&0&0&0&0\\ 0&0&0&0&0&0&0&0&0&0&0&0\\ 0&0&0&0&0&0&0&0&0&0&0&0\\ 0&0&0&0&0&0&0&0&1&0&0&0\\ 0&0&0&0&0&0&0&0&0&0&0&0\\ 0&0&0&0&0&0&0&0&0&0&0&0\\ 0&0&0&0&0&0&0&0&0&0&0&1\\ 0&0&0&0&0&0&0&0&0&0&0&0 \end{pmatrix}.$$

References

[1] Kelley BP, Sharan R, Karp RM, Sittler T, Root DE, Stockwell BR, Ideker T (2003) Conserved pathways within bacteria and yeast as revealed by global protein network alignment. *Proc Natl Acad Sci USA* **100**(20):11394–11399.

[2] $http://www.cytoscape.org/plugins1.php$

[3] Li Z, Zhang S, Wang Y, Zhang XS, Chen L (2007) Alignment of molecular networks by integer quadratic programming. *Bioinformatics* **23**(13):1631–1639.
